# Computational modelling of attentional bias towards threat in paediatric anxiety

**DOI:** 10.1111/desc.13055

**Published:** 2020-11-23

**Authors:** Abigail Thompson, Nikolaus Steinbeis

**Affiliations:** ^1^ Department of Clinical, Educational and Health Psychology UCL London UK

**Keywords:** anxiety, attention bias towards threat

## Abstract

Computational modelling can be used to precisely characterize the cognitive processes involved in attentional biases towards threat, yet so far has only been applied in the context of adult anxiety. Furthermore, studies investigating attentional biases in childhood anxiety have largely used tasks that conflate automatic and controlled attentional processes. By using a perceptual load paradigm, we separately investigate contributions from automatic and controlled processes to attentional biases towards negative stimuli and their association with paediatric anxiety. We also use computational modelling to investigate these mechanisms in children for the first time. In a sample of 60 children (aged 5‐11 years) we used a perceptual load task specifically adapted for children, in order to investigate attentional biases towards fearful (compared with happy and neutral) faces. Outcome measures were reaction time and percentage accuracy. We applied a drift diffusion model to investigate the precise cognitive mechanisms involved. The load effect was associated with significant differences in response time, accuracy and the diffusion modelling parameters drift rate and extra‐decisional time. Greater anxiety was associated with greater accuracy and the diffusion modelling parameter ‘drift rate’ on the fearful face trials. This was specific to the high load condition. These findings suggest that attentional biases towards fearful faces in childhood anxiety are driven by increased perceptual sensitivity towards fear in automatic attentional systems. Our findings from computational modelling suggest that current attention bias modification treatments should target perceptual encoding directly rather than processes occurring afterwards.

## Graphical abstract

Using drift diffusion modelling, we investigated the contributions of automatic and controlled attentional processes to attentional biases towards negative stimuli and their association with paediatric anxiety in children aged 5‐11. Higher anxiety was associated with higher accuracy (a) and the diffusion modelling parameter ‘drift rate’ (b). This was specific to the trials showing fearful faces in the high load condition. These findings suggest that attentional biases towards fearful faces in childhood anxiety are driven by increased perceptual sensitivity towards fear in automatic attentional systems.

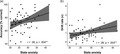

**Research highlights**

- This study provides the first application of computational modelling to attentional biases towards threat and its relationship to anxiety in children.
- By using a perceptual load paradigm, we separately investigate contributions from automatic and controlled processes to attentional biases.
- We report a specific relationship between anxiety and the computational modelling parameter ‘drift rate’, suggesting an increased speed of information uptake, before a perceptual decision is made.
- These results have implications for current attention bias modification treatments, suggesting that these should target perceptual encoding directly rather than processes that occur afterwards.

## 1 INTRODUCTION

Attentional biases towards threat‐related stimuli including fearful faces (Cisler & Koster, 2010) are a hallmark of anxiety disorders. Such biases in attention are a potential target for clinical treatments (Hakamata et al., 2010), may arise in infancy and are thought to play a causative role in the development and maintenance of anxiety symptoms (Pérez‐Edgar et al., 2010; Van Bockstaele et al., 2014). Despite the putative etiological and clinical significance of attentional biases in anxiety, the majority of studies investigating this have been conducted in adults. As anxiety disorders emerge early, constitute a major childhood psychiatric problem (Kessler et al., 2005) and are highly persistent into adulthood it is critical to investigate how these mechanisms emerge and change in child development (Lee et al., 2014).

At any given moment, attentional selection is the product of a dynamic interplay between stimulus‐driven (automatic, involuntary) and goal‐driven (controlled, voluntary) processes (Corbetta & Shulman, 2002; Serences et al., 2005). Automatic (or ‘bottom up’) attentional processes can occur outside conscious awareness (Liddell et al., 2005) and bias attention towards salient environmental stimuli, based on sensory features (Buschman & Miller, 2007). Controlled attentional processes enable attention to remain in line with the demands of the current situation, such as completing a task (Petersen & Posner, 2012). In this way, controlled attentional processes are sensitive to contextual factors such as current goals (Yantis, 2000).

The distinction between automatic and controlled attentional systems is important developmentally because neural systems supporting automatic and controlled attentional processing display distinct developmental trajectories (Casey et al., 2008). Automatic attentional mechanisms, including the influence of emotion on attention, develop early, with studies suggesting emotional influences on attention in infancy (Peltola et al., 2009). In contrast, attentional control develops later in childhood, supported by the development of the prefrontal cortex (Posner et al., 2014).

Alterations to both automatic and controlled processes may contribute towards attentional biases towards threat in anxiety. The Attentional Control Theory of anxiety posits that stimulus‐driven processing is prioritised and top‐down control over attention is weakened (Eysenck et al., 2007), suggesting the overall balance between the two systems is altered. In line with this, both heightened amygdala (McClure et al., 2007; Monk et al., 2008) and reduced prefrontal activity (Bishop et al., 2007) have been reported during processing of emotional faces. Studies that have investigated these mechanisms in childhood suggest that the development of controlled and automatic processes may be etiologically meaningful. For instance, the normative attentional bias towards fearful faces present in infancy (Peltola et al., 2009) is subsequently down‐regulated over the course of development in children who do not go on to develop anxiety, with abnormality in this regulatory mechanism in children that do (Dudeney et al., 2015; Fu et al., 2017).

However, developmental studies are not always consistent and some studies report an absence of attentional biases in children with anxiety (Britton et al., 2012). Conflicting findings may relate to methodological limitations (Rodebaugh et al., 2016; Schmukle, 2005), pointing to the need to use novel methods and new tasks to investigate these processes (Hauser et al., 2019; Price et al., 2019). The most commonly used tasks, the Dot‐Probe and Emotional Stroop (Bar‐Haim et al., 2007; Dudeney et al., 2015) conflate controlled and automatic attentional processes and thus do not allow these mechanisms to be investigated separately. One paradigm that allows separate investigation of automatic and controlled attentional processes is the perceptual load paradigm (Lavie, 1995; Lavie & Tsal, 1994). The paradigm uses varying ‘load’ conditions, which either fully or partially occupy perceptual resources. In a so‐called ‘high load’ condition, perceptual resources are fully occupied, causing early attentional selection to occur. This is driven by perceptual features of the stimulus and in this way is considered to be ‘bottom‐up’, or automatic (Theeuwes et al., 2004). Due to this, distractors are not perceived, indicated by low levels of distractor interference (Lavie, 1995). In a so‐called ‘low load’ condition, characterised by a less complex perceptual display, perceptual resources are not fully occupied. This causes ‘attentional spillover’, leading to the perception of irrelevant information (such as distractors). In this condition, attentional selection occurs late in perceptual processing, requiring top‐down control to avoid distraction by irrelevant stimuli. These two conditions therefore allow a dissociation between early automatic and later controlled processes in attentional biases. Few studies have used the perceptual load paradigm in anxiety and so far only in adults. Those that do, have consistently reported increased distraction in adults in the high load condition (Bishop et al., 2007; Moriya & Tanno, 2010; Sadeh & Bredemeier, 2011; Soares et al., 2015). Whether this is also the case in childhood anxiety is unknown.

A second methodological factor that may influence current findings is that current tasks looking at attentional biases to threat are hampered by methodological limitations (Rodebaugh et al., 2016; Schmukle, 2005), which may partly relate to the predominant use of average reaction time measures which conflate multiple cognitive processes (Enkavi et al., 2019). Recent developments in computational modelling have led to analysis approaches that are more refined and demonstrate improved reliability in the context of anxiety (Price et al., 2019). An example of this is the drift diffusion model (Ratcliff & McKoon, 2008), which uses the response time distributions and correct/incorrect performance to produce parameters reflecting separate components of a speeded response and allow parsing of distinct processes of attentional biases. Of particular interest to the study of attentional biases in anxiety are the drift diffusion model measures, drift rate (*V*) and non‐decisional process (*t0*). *V* reflects the speed of uptake of information from the stimulus and reflects perceptual processing speed and task difficulty. *V* may be sensitive to perceptual biases (Leong et al., 2019) and is influenced by affective state (White et al., 2010) and has been linked to anxiety in recent studies (Aylward et al., 2019). *T0* reflects encoding processes occurring before and after the decision, which may reflect eye movements (Price et al., 2019) and orienting to a stimulus, as well as motor execution.

Therefore, in the present study we sought to conduct the first investigation of the role of automatic and controlled processes in attentional bias to threat in state anxiety in a developmental sample aged 5‐11 years, by applying computational modelling to a perceptual load paradigm using emotional stimuli. We hypothesized that high state anxious participants would be distracted by fearful faces, and that this would be specific for the high load condition, in line with alterations in processing, respectively.

## 2 MATERIALS AND METHODS

### 2.1 Participants

One hundred and eight 5.49‐11.36 year‐olds (Mean age = 7.76, SD = 1.71) participated in the experiment. Participants were recruited from a regular education school based in Greater London. Tasks were completed in a classroom as part of a battery of tests. Prior to their children's participation, informed consent was obtained for all participants and the study was approved by the internal review and ethics board of University College London. Children who performed with an overall accuracy less than 67% on the Emotional Symbol Search task were removed from the analysis, based on previous studies investigating the load effect in an age‐group similar to ours (Huang‐Pollock et al., 2002). The final number of participants was 60 (Mean age = 8.04 which did not differ significantly in age from the original group; t(120) = 1.01, *p* = .32 SD = 1.74; range 5.50‐11.36 years).

### 2.2 Measures

#### 2.2.1 Emotional symbol search

The task was presented on a portable laptop PC using MATLAB. The task was adapted from Bishop (2007). Due to reports that children may have difficulty perceiving letters during a perceptual load task (Huang‐Pollock et al., 2002), we adapted the task to use symbols rather than letters (McDermott et al., 2007). Other than the target stimuli used, we replicated the task from the description of Bishop et al. (2007). On each trial, an array of six symbols comprising different single colour shapes was superimposed on a task‐irrelevant unfamiliar face for 200 ms. The shapes included a circle, triangle, arrow, star, cross, square and a square rotated 45°. The field of view was consistent with that of Jenkins et al. (2005). The task was to decide whether the array contained a circle or a triangle, the appearance of which occurred randomly and equiprobably. A target was present on every trial. In the ‘high load’ condition, a triangle or circle appeared surrounded by five different shapes. In the ‘low load’ condition, the array consisted of six triangles or circles. Participants were instructed to press the ‘X’ key if they saw a circle, and press the ‘N’ key if they saw a triangle. Participants were instructed to respond as quickly as possible. Total trial length was 3 seconds and a fixation cross appeared for 1 second in between each trial. Participants wore headphones and received feedback for incorrect or omitted responses in the form of a computer tone (Huang‐Pollock et al., 2002). No tone was provided for correct trials. The presented faces were neutral, happy or fearful. Happy and fearful faces were presented pseudo‐randomly, and were interspersed by neutral faces. There were two variants of the task; one using child emotional faces (LoBue & Thrasher, 2014), and the other using adult emotional faces (Lundqvist et al., 1998). All faces were edited to remove hair and were transformed to greyscale. Due to technical complications, the total number of trials experienced by participants varied (range = 101‐356 trials, median = 203, mean = 220.88, standard deviation = 67.33).

#### 2.2.2 State‐trait anxiety for children

As we were seeking to understand how anxiety interacts with task performance, children were asked to rate how they felt at the time of testing. State anxiety symptoms were measured using the “State” component of the State‐Trait Anxiety inventory for children (STAI‐C) (Spielberger, 1973). There were 20 items, each of which had three options (for example, “I feel…” “very calm”, “calm” or “not calm”). Children did not complete the “trait” component of the measure. A greater score on the STAI‐C corresponds to greater levels of anxiety. The STAI‐C has high internal consistency and high test–retest reliability (Spielberger and Gorsuch, 1983).

#### 2.2.3 Data analysis and processing

For the Emotional Symbol Search task, the dependent variables were accuracy and reaction time across correct trials. Responses faster than 100 ms were removed from the analysis (as in previous studies, e.g. Huang‐Pollock et al., 2005).

#### 2.2.4 Drift‐diffusion modelling (DDM)

Fast‐dm software was used for calculation of DDM measures drift rate (V), extradecisional time (t0) and other measures, based on the trial‐level reaction time and correctness (0 = incorrect, 1 = correct) data for each participant (Voss & Voss, 2007). The Kolmogorov–Smirnov method was used to calculate drift rate (V), extradecisional time (t0), decisional threshold separation (a), differences in response execution (d), and three variability measures (st0, sv, sz). Final parameter estimation was guided by the following model comparisons.

Following recommended guidelines (Voss et al., 2015), we first allowed all model parameters to vary freely between conditions (high and low load; high load fearful, neutral, happy faces; low load fearful, neutral, happy faces), and compared this with constraining all parameters to be equal across conditions. Whilst the model fits were good in both cases, there was a marginal increase in the mean model fit when the model parameters were able to vary between conditions (e.g. Kolmogorov–Smirnov test p‐value improved from mean = .95 to .98), in line with recommendations. A limitation of this approach is that by separately modelling the specific task conditions we reduce the total trial numbers included in the model, which may be considered towards the lower end of recommended trial numbers. However, a recent systematic investigation of the impact of trial number on drift diffusion model parameter recovery demonstrated robust parameter estimation with small trial numbers (Lerche et al., 2017). Based on this we selected the option of modelling all parameters separately across task conditions.

Next, we assessed the impact on model fits of holding variability parameters (sv, st0, sz) at 0 or allowing them to be estimated in the data, following recommendations that holding the intertrial variability parameters stable may improve estimation of other parameter values (Lerche et al., 2017). We found that holding these parameters at 0 marginally reduced the mean fit (Kolmogorov–Smirnov test *p*‐value from mean = 0.98 to 0.97).

Finally, we tested the recommendation that holding the percentage of contaminants at 0 and a priori decisional bias at 0.5 would lead to a better model fit, and found this to be supported (Kolmogorov–Smirnov test *p*‐value 0.98).

Therefore, the final model consisted of 7 model parameters, applying a "correctness" model to separately to the different trials (high and low load; high load fearful, neutral, happy faces; low load fearful, neutral, happy faces). Percentage of contaminants (p) was set to 0 and a priori decisional bias (zr) was set to 0.5 (Voss et al., 2013). Model fit was assessed with the Kolgoromov–Smirnov tests. Data were excluded from the analysis where model fit did not reach > 0.6. Correlation heat‐maps between drift diffusion model parameters (drift rate, *V* and non‐decisional processes, *t0*) and canonical measures (mean response time and accuracy) are presented in Supplementary Figures S5 and S6.

#### 2.2.5 Statistical analyses

We used R (R Core Team, 2012) and *lme4* to perform a linear mixed effects analysis investigating the relationship between load, age (and the interaction between load and age) for each of the dependent variables (accuracy, RT, *V* and t0). Separate analyses were run investigating each of these dependent variables. For each, we entered load and age as fixed effects, including the interaction term. Number of trials was also included as a fixed effect in order to control for this. Random intercepts were included for subjects and random slopes for load. When fitting the mixed‐effects model with V and T0 as the dependent variables, the model failed to converge when random intercepts were included for subjects and random slopes for load. We therefore kept the intercept for subjects but removed the slope for load.

Continuous predictors were mean centred. Residual plots were visually inspected and did not reveal any deviations from homoscedasticity or normality. Likelihood ratio tests were used, comparing the full model to a model with the effect in question removed, to obtain p‐values. Two‐tailed Pearson's r correlations were used to test the relationship between state anxiety measures and mean performance on the Emotional Symbol Search task, for the high and low load conditions separately, controlling for number of trials.

## 3 RESULTS

### 3.1 Load

**Figure 1:**
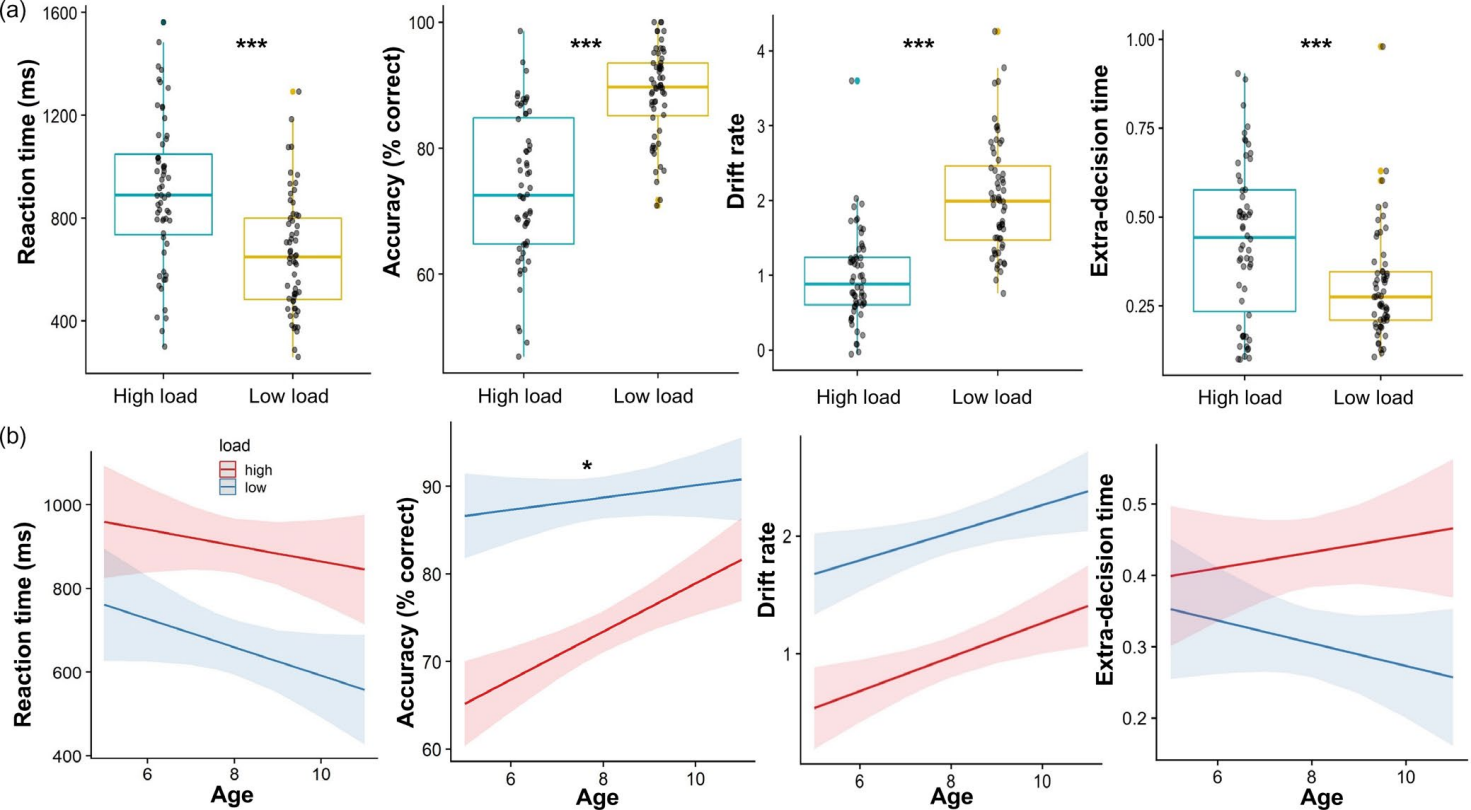
(a) Differences between the high and low load conditions on reaction time, accuracy, drift rate and extra‐decision time, (b) Relationship with age (in years) across high and low load. *p < 0.05, ***p < 0.001

Linear mixed effects analyses were conducted for RT, accuracy, and the diffusion measures *V* and t0, with perceptual load (low/high) and age as predictor variables (and the interaction between load and age), and number of trials controlled (see Supplementary Materials Table S1 for full model parameters including confidence intervals, Figure 1).

#### 3.1.1 Accuracy

There was a significant interaction between age and load (X^2^(1) = 3.96, *p* = <0.05). Follow‐up comparisons revealed a positive relationship between age and accuracy in the high load condition (r = .39, *p* = .002 CI [0.14, 0.58]) but not in the low load condition (*p* > .05). The main effect of load was significant (X^2^(1) = 75.53, *p* = <0.001), with significantly greater accuracy in the low load (*M* = 0.89; SD = 0.32) compared to the high load condition (*M* = 0.75; SD = 0.44).

#### 3.1.2 Reaction times

The interaction between age and load was not significant. The analysis revealed a significant main effect of load (X^2^(1)=65.03, *p* = <0.001), with significantly greater reaction times in the high load (*M* = 918.61; SD = 488.94) condition compared with the low load (*M* = 668.07; SD = 390.49) condition. There was also a significant main effect of age (X^2^(1) = 23.70, *p* = <0.001), reflecting that children responded faster with increasing age.

#### 3.1.3 Drift diffusion model measures

The main effect of load on *V* was significant (X^2^(1) = 62.09, *p* = <0.001), with significantly greater *V* in the low load condition (*M* = 2.03; SD = 0.76) than in the high load condition (*M* = 0.98; SD = 0.62). There was a significant main effect of age on *V* (X^2^(1)=10.00, *p* = 0.002), reflecting that *V* increases with age in both load conditions. There was also a significant main effect of load on t0 (X^2^(1) = 22.38, *p* < 0.001), with significantly greater t0 in the high load (*M* = 0.43; SD = 0.22) compared with the low load condition (*M* = 0.30; SD = 0.15). No other main effects or interactions were significant.

### 3.2 Relationship between load, emotional distractors and anxiety

To study the relationship between performance on the perceptual load task during the different distractor faces and state anxiety we conducted a series of correlations investigating this, across loads. Given the previous literature we anticipated effects during the presentation of fearful faces. Two children did not complete the STAI‐C due to time constraints during testing, therefore the number of participants included in this analysis was 58. Means and standard deviations for RT and accuracy by perceptual load and expression are presented in Supplementary Materials Table S2.

#### 3.2.1 Accuracy

**Figure 2:**
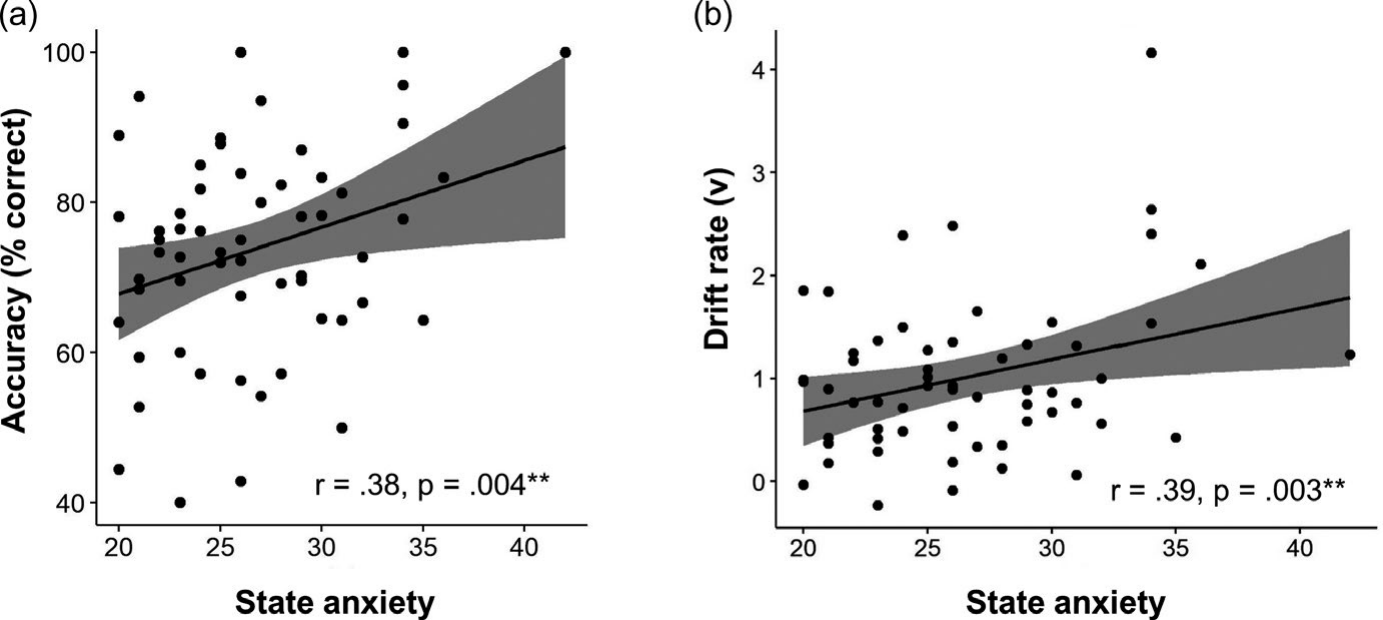
Relationship between state anxiety scores and mean accuracy scores (left) and drift rate (right) on the ‘fearful faces’ trials in the high load condition only, when controlling for age, number of trials and accuracy performance on the fearful faces trials, low load condition

There was a positive correlation between mean accuracy and state anxiety scores on trials where fearful faces were presented in the high load condition, (r = 0.30, *p* = 0.02 CI [0.05, 0.52]), but not in the low load condition (r = 00.02, *p* > 0.05 CI [−0.24 0.27]). This remained significant when age (r = 0.34, *p* = 0.009 CI [0.09 0.55]) and performance on the fearful face trials of the low load condition were included as covariates (r = 0.37, *p* = 0.005 CI [0.13 0.57]). This finding was specific to fearful faces: there were no significant correlations between state anxiety and accuracy performance on either the happy or neutral trials. To further investigate the specificity of this relationship we also conducted a partial correlation controlling for accuracy in the low load fearful face trials and accuracy on the other facial expression conditions (high load), as well as number of trials, and the correlation remained significant (r = 0.31, *p* = .02 CI [0.06 0.52]) (Figure 2a). Graphs of the relationship between accuracy and state anxiety for the other facial expressions and the low load condition are included in the supplementary materials (Supplementary Figures S1‐S4).

#### 3.2.2 Drift diffusion model

To further investigate the relationship between state anxiety and performance on the fearful face trials, we conducted a correlation between the drift diffusion measures *V* and t0 and state anxiety on the fearful face trials. There was a significant correlation between state anxiety and drift rate on the fearful face trials of the high load condition (r = 0.31, *p* = 00.02 CI [0.06 0.52]), but not in the low load condition (r = −0.06, *p* > 0.05 CI [−0.31 0.20]). *Z* observation analysis for dependent correlations revealed these correlations to be significantly different (z = 2.04, *p* = 0.04). The relationship in the high load condition remained significant when age (r = 0.36, *p* = .006 CI [0.12 0.56]) and performance on the fearful face trials of the low load condition (r = 0.40, *p* = 0.002, CI [0.16 0.59]) were included as covariates (Figure 2b). Correlations with the other facial expressions did not reach significance. Furthermore, when controlling for *V* in the low load fearful face trials and on the other facial expression conditions (high load), as well as number of trials, the correlation remained significant, (r = 0.27, *p* = <0.05, CI [0.02 0.49]). There were no significant correlations between state anxiety and t0.

#### 3.2.3 Reliability

To test the internal consistency of the perceptual load task, we conducted a split‐half reliability test on reaction times. We found that all our main effects have an internal consistency greater than r = 0.58, <0.001, suggesting high internal consistency.

#### 3.2.4 Sensitivity analyses

We conducted sensitivity analyses, with minimum number of trials used for all subjects and on the participants that were excluded from the main analysis due to performing with overall accuracy of less than 67% (*N* = 48), in order to test the sensitivity of our findings. When using minimum trial numbers across subjects we found that the reported main effects hold. When conducting the analysis on the participants excluded on the basis of performance accuracy, we found that there was a significant load effect (accuracy, reaction time and V) and significant effect of age (reaction time, t0). There were no significant correlations between state anxiety and performance on fearful face trials. The full results from these analyses are included in the supplementary material, under the section titled ‘Sensitivity Analyses’.

## 4 DISCUSSION

We used a perceptual load paradigm and applied computational modelling to investigate the processes underlying attentional biases towards threat in paediatric anxiety. Our main finding was that state anxiety was associated with increased accuracy on fearful face trials in the high load condition only. Application of diffusion modelling further clarified this association as we report that greater state anxiety was also associated with faster speed of information processing, indicated by drift rate, on the fearful face trials. Below we discuss how our findings shed novel light on the etiology of anxiety and their implications for clinical treatments.

Increased attention towards threat has been reported across a range of anxiety sub‐types, tasks and ages (Bar‐Haim et al., 2007), and thus our findings are in line with this. However, compared to previous studies that have used tasks which conflate automatic and controlled processes, our study lends novel insight into the distinct contribution of these processes to attentional biases in childhood anxiety. Our specific effect in the high load condition is informative given the experimental manipulations of this condition. The load theory states that in the high load condition, attentional selection occurs early due to the attentional resources being fully occupied, causing perception of distractors to be less likely (Lavie, 1995). This is stimulus driven, rather than driven by control processes, and in this way is considered to tap into automatic attentional processes (Theeuwes, 2010). In this regard, our results suggest that state anxiety may alter early, automatic attentional processes. This is in line with other behavioural studies that report increased distraction under high load in adults with trait and social anxiety (Sadeh & Bredemeier, 2011; Soares et al., 2015) and suggest that a similar mechanism operates in paediatric anxiety.

Using computational modelling enables us to characterize the cognitive processes more precisely (Hauser et al., 2019). By separating the time‐course of a speeded response into components that occur before and after a decision is made, drift diffusion modelling enables separate investigation of the speed of perceptual encoding, known as drift rate. We report that heightened anxiety is associated with a faster speed of processing on trials where fearful faces were shown, in the high load condition. This was not the case either for trials with another emotional facial expression (happy) or for trials with neutral faces. As drift rate can be conceptualised as the amount of information extracted from the stimulus and is akin to information processing, this suggests enhanced perceptual processing of threat and may reflect a perceptual bias (Leong et al., 2019). Previous diffusion modelling studies have shown that affect influences perceptual encoding (Roberts & Hutcherson, 2019). Our findings may suggest that high anxiety causes individuals to give greater weight to negative affect in their perceptual processing, and that this can lead to a performance advantage (Gasper & Clore, 1998), in line with the association with increased accuracy reported in the present study.

Attention bias modification has often been used as a clinical treatment for childhood anxiety, however, it has produced highly inconsistent results (McNally, 2019). Attention‐bias treatments primarily target attentional processes that occur after perceptual encoding has already occurred (such as targeting attention away from threat) (Mogg & Bradley, 2016). Our finding of heightened perceptual *encoding* suggests treatments should target this earlier occurring perceptual mechanism. As perceptual encoding may relate to motivational factors (Leong et al., 2019), such an approach may be in line with interventions that target the interpretation of stimuli, as opposed to directing attention away from stimuli after they have already been perceived (Mogg & Bradley, 2016). The increased level of detail provided by our modelling approach may also help to shed light on inconsistencies within the developmental literature, in which contrasting findings have been reported on the basis of mean reaction time measures.

Fearful faces, compared with happy or neutral, specifically activate a fast‐acting route to the amygdala (Méndez‐Bértolo et al., 2016). Because high load is thought to engage automatic attentional processes, the presentation of fearful faces may be associated with fast activation of the amygdala, which may then bias subsequent processing in both occipital and prefrontal regions, leading to better discrimination performance on the task. This may lead to a ‘facilitatory’ effect of the fearful faces. This is consistent with reports of enhanced processing of emotional stimuli (Pourtois et al., 2013), suggesting that increased levels of state anxiety may amplify this normative perceptual effect. Such a facilitatory effect could also occur in the low load condition, yet we did not find this in the current study. Since low load engages automatic attentional processes less, it is possible that any initial fast‐acting amygdala activation is subsequently modulated by contextual and goal‐related factors, once the signal reaches the prefrontal cortex (for example, due to the irrelevance of the faces to performance on the task) (Corbetta & Shulman, 2002).

We also found that reaction time performance on both the high and low load conditions improve with age over mid‐late childhood, however there was a specific improvement in accuracy performance in the high load condition with age. This runs counter to the findings of Huang‐Pollock et al. (2002), who report that children aged 7‐12 performed as well as adults on the high load condition, but showed continued development on the low load condition. Huang‐Pollock et al. (2002) explain their findings as supporting the hypothesis that high load taps into earlier developing, automatic attentional systems, whilst, in contrast, because the low load condition is thought to require attentional control, this may develop later. In contrast with all previous developmental studies investigating load (Couperus, 2011; Huang‐Pollock et al., 2002, 2005), we used an adapted load task, which used symbols instead of letters as target stimuli, which may be more appropriate for this age range (McDermott et al., 2007). Our findings suggest that performance on both load conditions continues to develop in mid to late childhood and thus, that more bottom‐up automatic attentional processes may also undergo continued development. Indeed, whilst the distinction between ‘automatic’ and controlled processing in line with the perceptual load task is supported by numerous lines of evidence (Maylor & Lavie; Bishop, 2007; Bishop, 2009; Handy & Mangun, 2000; Handy & Soltani 2001), we cannot conclusively claim that face detection is limited to the automatic component of attention. The developmental findings that we present may suggest that these two conditions may not map as clearly onto the distinct attentional systems, as suggested by the original theory. Future studies could confirm this by using brief stimulus presentations or by manipulating the response time window, in order to isolate the automatic component of attention.

An important consideration is whether the results presented here may be explained by other factors such as arousal or recognition ability. Emotional facial expressions have greater subjective arousal ratings than neutral, but reports differ regarding the arousal levels of fearful and happy faces (Sato & Yoshikawa, 2007; Springer et al., 2007; Goeleven et al., 2008; Mancini et al., 2013). Some studies report no significant differences between arousal levels of fearful and happy faces (Springer et al., 2007; Goeleven et al., 2008). Other studies, including in children aged 8‐11 which overlaps with the age‐group presented here (Mancini et al., 2013), report that happy faces are associated with significantly greater levels of arousal (Sato & Yoshikawa, 2007). This suggests that the specific effect of fearful faces that we report in the current study is unlikely to be due to baseline differences in arousal levels across the face stimuli presented.

However, there may be a specific interaction between arousal, valence and anxiety. For instance, negative stimuli of low arousal have been found to be associated with better task performance in anxious individuals (Sussman et al., 2013). This effect is not found for stimuli of positive valence or negative high arousal. Stimuli that are considered high arousal include those that signal a direct threat (i.e. a gun pointing directly at the viewer). In contrast, fearful facial expressions indicate the presence of threat in the environment, but its relevance to the viewer is not as immediate, and thus, compared with stimuli containing more direct threats, fearful faces may be considered low arousal negative stimuli. This suggests that in the current study, anxious individuals might have a performance advantage, specifically when processing fearful faces, due to these faces being negative low‐arousal stimuli. Further studies are needed to determine whether the reported effect is specific to fearful faces per se, or whether a similar performance advantage would be present for other negative low‐arousal stimuli, such as negative scenes.

Fearful faces have been shown to be less easy to recognize than other expressions, including happy and neutral (Gross & Ballif, 1991; Rapcsak et al., 2000; Herba & Phillips, 2004; Rodger et al., 2018). It has been suggested that the threat processing system specifically promotes more processing of ambiguous stimuli, in order to determine whether or not they constitute a threat (Davis & Whalen, 2001). According to this interpretation, state anxiety could have provided a performance advantage on fearful face trials specifically because, as ambiguous stimuli, the fearful faces may have led to more attentive processing. Importantly however, when considering the effects both of arousal and recognition differences, it is of note that the performance advantage that we report (in terms of accuracy performance, for state anxiety on the fearful face trials) was present only in the high load condition of the task, and not the low load condition. In relation to recognition abilities, it is unclear why such an effect would not also be present in the low load condition. If the results are explained by arousal, this would suggest that the high load condition may be associated with less arousal than the low load condition. As the high load condition is considerably more demanding (indexed by greater error rates), we think this is implausible.

In the present study, we used an accuracy threshold of 67% for the main analysis. Such a threshold was deemed necessary to ensure that sufficient attention was paid to the task, and is in line with previous studies that have used a similar perceptual load task in children of a similar age to ours (Huang‐Pollock et al., 2002). However, it is important to note that the specific relationship between anxiety and performance on fearful face trials was not found when a sensitivity analysis was run on the excluded participants.

The present study is the first to investigate attentional processes in anxiety in a developmental cohort, with a perceptual load task adapted for use with children and, to our knowledge, is the first study to apply a computational model to these processes in paediatric anxiety. There was a specific ‘facilitatory’ effect of state anxiety on accuracy performance on the high load condition trials when fearful faces were presented, and these trials were also associated with heightened information uptake, which may suggest increased perceptual processing. These findings provide novel insight into the developmental processes associated with perceptual biases in anxiety and inform clinical treatments which seek to modify attentional processes in anxiety.

## CONFLICT OF INTEREST

None.

## Supporting information

Supplementary Material

## Data Availability

The data that support the findings of this study are available from the corresponding author upon reasonable request.

## References

[desc13055-bib-0001] Aylward, J. , Hales, C. , Robinson, E. , & Robinson, O. J. (2019). Translating a rodent measure of negative bias into humans: The impact of induced anxiety and unmedicated mood and anxiety disorders. Psychological Medicine, 50(2), 237–246. 10.1017/S0033291718004117 30683161 PMC7083556

[desc13055-bib-0002] Bar‐Haim, Y. , Lamy, D. , Pergamin, L. , Bakermans‐Kranenburg, M. J. , & van IJzendoorn, M. H. (2007). Threat‐related attentional bias in anxious and nonanxious individuals: A meta‐analytic study. Psychological Bulletin, 133(1), 1–24. 10.1037/0033-2909.133.1.1 17201568

[desc13055-bib-0003] Bishop, S. J. (2009). Trait anxiety and impoverished prefrontal control of attention. Nature Neuroscience, 12(1), 92–98. 10.1038/nn.2242 19079249

[desc13055-bib-0004] Bishop, S. J. , Jenkins, R. , & Lawrence, A. D. (2007). Neural processing of fearful faces: Effects of anxiety are gated by perceptual capacity limitations. Cerebral Cortex, 17(7), 1595–1603. 10.1093/cercor/bhl070 16956980

[desc13055-bib-0005] Britton, J. C. , Bar‐Haim, Y. , Carver, F. W. , Holroyd, T. , Norcross, M. A. , Detloff, A. , …, Pine, D. S. (2012). Isolating neural components of threat bias in pediatric anxiety. Journal of Child Psychology and Psychiatry and Allied Disciplines, , 53(6), 678–686. 10.1111/j.1469-7610.2011.02503.x PMC3354023 22136196

[desc13055-bib-0006] Buschman, T. J. , & Miller, E. K. (2007). Top‐down versus bottom‐up control of attention in the prefrontal and posterior parietal cortices. Science, 315(5820), 1860–1862. 10.1126/science.1138071 17395832

[desc13055-bib-0007] Casey, B. J. , Jones, R. M. , & Hare, T. A. (2008). The adolescent brain. Annals of the New York Academy of Sciences, 10.1196/annals.1440.010 PMC2475802 18400927

[desc13055-bib-0008] Cisler, J. M. , & Koster, E. H. W. (2010). Mechanisms of attentional biases towards threat in anxiety disorders: An integrative review. Clinical Psychology Review, 30(2), 203–216. 10.1016/j.cpr.2009.11.003 20005616 PMC2814889

[desc13055-bib-0009] Corbetta, M. , & Shulman, G. L. (2002). Control of goal‐directed and stimulus‐driven attention in the brain. Nature Reviews Neuroscience, 3(3), 201–215. 10.1038/nrn755 11994752

[desc13055-bib-0010] Couperus, J. W. (2011). Perceptual load influences selective attention across development. Developmental Psychology, 47(5), 1431–1439. 10.1037/a0024027 21688896

[desc13055-bib-0011] Davis, M. , & Whalen, P. J. (2001). The amygdala: Vigilance and emotion. Molecular Psychiatry, 6(1), 13–34. 10.1038/sj.mp.4000812 11244481

[desc13055-bib-0012] Dudeney, J. , Sharpe, L. , & Hunt, C. (2015). Attentional bias towards threatening stimuli in children with anxiety: A meta‐analysis. Clinical Psychology Review, 40, 66–75. 10.1016/j.cpr.2015.05.007 26071667

[desc13055-bib-0068] Enkavi, A. Z. , Eisenberg, I. W. , Bissett, P. G. , Mazza, G. L. , MacKinnon, D. P. , Marsch, L. A. , & Poldrack, R. A. (2019). Large‐scale analysis of test–retest reliabilities of self‐regulation measures. Proceedings of the National Academy of Sciences, 116(12), 5472–5477. 10.1073/pnas.1818430116 PMC6431228 30842284

[desc13055-bib-0013] Eysenck, M. W. , Derakshan, N. , Santos, R. , & Calvo, M. G. (2007). Anxiety and cognitive performance: Attentional control theory. Emotion, 7(2), 336–353. 10.1037/1528-3542.7.2.336 17516812

[desc13055-bib-0014] Fu, X. , Taber‐Thomas, B. C. , & Pérez‐Edgar, K. (2017). Frontolimbic functioning during threat‐related attention: Relations to early behavioral inhibition and anxiety in children. Biological Psychology. Elsevier B.V., 122, 98–109. 10.1016/j.biopsycho.2015.08.010 PMC4779741 26325222

[desc13055-bib-0015] Gasper, K. , & Clore, G. L. (1998). The persistent use of negative affect by anxious individuals to estimate risk. Journal of Personality and Social Psychology, 74(5), 1350–1363. 10.1037/0022-3514.74.5.1350 9599448

[desc13055-bib-0016] Goeleven, E. , De Raedt, R. , Leyman, L. , & Verschuere, B. (2008). The Karolinska directed emotional faces: A validation study. Cognition and Emotion, 22(6), 1094–1118. 10.1080/02699930701626582

[desc13055-bib-0017] Gross, A. L. , & Ballif, B. (1991). Children’s understanding of emotion from facial expressions and situations: A review. Developmental Review, 11(4), 368–398. 10.1016/0273-2297(91)90019-K

[desc13055-bib-0018] Hakamata, Y. , Lissek, S. , Bar‐Haim, Y. , Britton, J. C. , Fox, N. A. , Leibenluft, E. , …, Pine, D. S. (2010). Attention bias modification treatment: A meta‐analysis toward the establishment of novel treatment for anxiety. Biological Psychiatry, 68(11), 982–990. 10.1016/j.biopsych.2010.07.021 20887977 PMC3296778

[desc13055-bib-0071] Handy T. C. , Mangun G. R. (2000). Attention and spatial selection: Electrophysiological evidence for modulation by perceptual load. Perception & Psychophysics, 62 (1), 175–186 10703265 10.3758/bf03212070

[desc13055-bib-0070] Handy T. C. , Soltani M. , Mangun G. R. (2001). Perceptual Load and Visuocortical Processing: Event‐Related Potentials Reveal Sensory‐Level Selection. Psychological Science, 12 (3), 213–218 11437303 10.1111/1467-9280.00338

[desc13055-bib-0019] Hauser, T. U. , Will, G.‐J. , Dubois, M. , & Dolan, R. J. (2019). Annual research review: Developmental computational psychiatry. Journal of Child Psychology and Psychiatry and Allied Disciplines, 60(4), 412–426. 10.1111/jcpp.12964 30252127

[desc13055-bib-0020] Herba, C. , & Phillips, M. (2004). Annotation: Development of facial expression recognition from childhood to adolescence: Behavioural and neurological perspectives. Journal of Child Psychology and Psychiatry and Allied Disciplines, 45(7), 1185–1198. 10.1111/j.1469-7610.2004.00316.x 15335339

[desc13055-bib-0021] Huang‐Pollock, C. L. , Carr, T. H. , & Nigg, J. T. (2002). Development of selective attention: perceptual load influences early versus late attentional selection in children and adults. Developmental Psychology, 38(3), 363–375. 10.1037/0012-1649.38.3.363 12005380

[desc13055-bib-0022] Huang‐Pollock, C. L. , Nigg, J. T. , & Carr, T. H. (2005). Deficient attention is hard to find: Applying the perceptual load model of selective attention to attention deficit hyperactivity disorder subtypes. Journal of Child Psychology and Psychiatry, 46(11), 1211–1218. 10.1111/j.1469-7610.2005.00410.x 16238668

[desc13055-bib-0023] Jenkins, R. , Lavie, N. , & Driver, J. (2005). Recognition memory for distractor faces depends on attentional load at exposure. Psychonomic Bulletin and Review, 12(2), 314–320. 10.3758/BF03196378 16082812

[desc13055-bib-0024] Kessler, R. C. , Walters, E. E. , Demler, O. , Chiu, W. T. (2005). Prevalence, severity, and comorbidity of 12‐month. Methods, 62, 617–627. 10.1001/archpsyc.62.6.617 PMC2847357 15939839

[desc13055-bib-0025] Lavie, N. (1995). Perceptual load as a necessary condition for selective attention. Journal of Experimental Psychology: Human Perception and Performance, 21(3), 451–468. 10.1037/0096-1523.21.3.451 7790827

[desc13055-bib-0026] Lavie, N. , & Tsal, Y. (1994). Perceptual load as a major determinant of the locus of selection in visual attention. Perception & Psychophysics, 56(2), 183–197. 10.3758/BF03213897 7971119

[desc13055-bib-0027] Lee, F. S. , Heimer, H. , Giedd, J. N. , Lein, E. S. , Sestan, N. , Weinberger, D. R. , & Casey, B. J. (2014). Adolescent mental health–Opportunity and obligation. Science, 346(6209), 547–549. 10.1126/science.1260497 25359951 PMC5069680

[desc13055-bib-0028] Leong, Y. C. , Hughes, B. L. , Wang, Y. , & Zaki, J. (2019). Neurocomputational mechanisms underlying motivated seeing. Nature Human Behaviour, 3(9), 962–973. 10.1038/s41562-019-0637-z 31263289

[desc13055-bib-0029] Lerche, V. , Voss, A. , & Nagler, M. (2017). How many trials are required for parameter estimation in diffusion modeling? A comparison of different optimization criteria. Behavior Research Methods. Behavior Research Methods, 49(2), 513–537. 10.3758/s13428-016-0740-2 27287445

[desc13055-bib-0030] Liddell, B. J. , Brown, K. J. , Kemp, A. H. , Barton, M. J. , Das, P. , Peduto, A. , …, Williams, L. M. (2005). A direct brainstem‐amygdala‐cortical “alarm” system for subliminal signals of fear. NeuroImage, 24(1), 235–243. 10.1016/j.neuroimage.2004.08.016 15588615

[desc13055-bib-0031] LoBue, V. , & Thrasher, C. (2014). The child affective facial expression (CAFE) set: Validity and reliability from untrained adults. Frontiers in Psychology, 5, 10.3389/fpsyg.2014.01532 PMC4285011 25610415

[desc13055-bib-0032] Lundqvist, D. , Flykt, A. , & Öhman, A. (1998). The Karolinska directed emotional faces (KDEF). 91(630), 2). CD ROM from Department of Clinical Neuroscience, Psychology section, Karolinska Institutet.

[desc13055-bib-0033] Mancini, G. , Agnoli, S. , Baldaro, B. , Ricci Bitti, P. E. , & Surcinelli, P. (2013). Facial expressions of emotions: Recognition accuracy and affective reactions during late childhood. Journal of Psychology: Interdisciplinary and Applied, 147(6), 599–617. 10.1080/00223980.2012.727891 24199514

[desc13055-bib-0034] McClure, E. B. , Monk, C. S. , Nelson, E. E. , Parrish, J. M. , Adler, A. , Blair, R. J. R. , …, Pine, D. S. (2007). Abnormal attention modulation of fear circuit function in pediatric generalized anxiety disorder. Archives of General Psychiatry, 64(1), 97. 10.1001/archpsyc.64.1.97 17199059

[desc13055-bib-0035] McDermott, J. M. , Pérez‐Edgar, K. , & Fox, N. A. (2007). Variations of the flanker paradigm: Assessing selective attention in young children. Behavior Research Methods, 39(1), 62–70. 10.3758/BF03192844 17552472

[desc13055-bib-0036] McNally, R. J. (2018). (2019) ‘Attentional bias for threat: Crisis or opportunity?’. Clinical Psychology Review, 69, 4–13. 10.1016/j.cpr.2018.05.005 29853421

[desc13055-bib-0037] Méndez‐Bértolo, C. , Moratti, S. , Toledano, R. , Lopez‐Sosa, F. , Martínez‐Alvarez, R. , Mah, Y. H. , …, Strange, B. A. (2016). A fast pathway for fear in human amygdala. Nature Neuroscience, 19(8), 1041–1049. 10.1038/nn.4324 27294508

[desc13055-bib-0038] Mogg, K. , & Bradley, B. P. (2016). Anxiety and attention to threat: Cognitive mechanisms and treatment with attention bias modification. Behaviour Research and Therapy, 87, 76–108. 10.1016/j.brat.2016.08.001 27616718

[desc13055-bib-0039] Monk, C. S. , Telzer, E. H. , Mogg, K. , Bradley, B. P. , Mai, X. , Louro, H. M. C. , …, Pine, D. S. (2008). Amygdala and ventrolateral prefrontal cortex activation to masked angry faces in children and adolescents with generalized anxiety disorder. Archives of General Psychiatry, 65(5), 568– 10.1001/archpsyc.65.5.568 18458208 PMC2443697

[desc13055-bib-0040] Moriya, J. , & Tanno, Y. (2010). Attentional resources in social anxiety and the effects of perceptual load. Cognition and Emotion, 24(8), 1329–1348. 10.1080/02699930903378503

[desc13055-bib-0041] Peltola, M. J. , Leppänen, J. M. , Mäki, S. , & Hietanen, J. K. (2009). Emergence of enhanced attention to fearful faces between 5 and 7 months of age. Social Cognitive and Affective Neuroscience, 4(2), 134–142. 10.1093/scan/nsn046 19174536 PMC2686224

[desc13055-bib-0042] Pérez‐Edgar, K. , Bar‐Haim, Y. , McDermott, J. M. , Chronis‐Tuscano, A. , Pine, D. S. , & Fox, N. A. (2010). Attention biases to threat and behavioral inhibition in early childhood shape adolescent social withdrawal. Emotion, 10(3), 349–357. 10.1037/a0018486 20515224 PMC3614079

[desc13055-bib-0043] Petersen, S. E. , & Posner, M. I. (2012). The attention system of the human brain: 20 years after. Annual Review of Neuroscience, 35(1), 73–89. 10.1146/annurev-neuro-062111-150525 PMC3413263 22524787

[desc13055-bib-0044] Posner, M. I. , Rothbart, M. K. , Sheese, B. E. , & Voelker, P. (2014). Developing attention: Behavioral and brain mechanisms. Advances in Neuroscience, 2014, 1–9. 10.1155/2014/405094 PMC4125572 25110757

[desc13055-bib-0045] Pourtois, G. , Schettino, A. , & Vuilleumier, P. (2013). Brain mechanisms for emotional influences on perception and attention: What is magic and what is not. Biological Psychology, 92(3), 492–512. 10.1016/j.biopsycho.2012.02.007 22373657

[desc13055-bib-0046] Price, R. B. , Brown, V. , & Siegle, G. J. (2019). Computational modeling applied to the dot‐probe task yields improved reliability and mechanistic insights. Biological Psychiatry, 85(7), 606–612. 10.1016/j.biopsych.2018.09.022 30449531 PMC6420394

[desc13055-bib-0047] Rapcsak, S. Z. , Galper, S. R. , Comer, J. F. , Reminger, S. L. , Nielsen, L. , Kaszniak, A. W. , …, Cohen, R. A. (2000). Fear recognition deficits after focal brain damage: A cautionary note. Neurology, 54(3), 575. 10.1212/WNL.54.3.575 10680785

[desc13055-bib-0048] Ratcliff, R. , & McKoon, G. (2008). The diffusion decision model: Theory and data for two‐choice decision tasks. Neural Computation, 20(4), 873–922. 10.1162/neco.2008.12-06-420 18085991 PMC2474742

[desc13055-bib-0069] R Core Team (2012). R: A language and environment for statistical computing, Vienna, Austria: R Foundation for Statistical Computing. http://www.R‐project.org/.

[desc13055-bib-0049] Roberts, I. D. , & Hutcherson, C. A. (2019). Affect and decision making: insights and predictions from computational models. Trends in Cognitive Sciences, 23(7), 602–614. 10.1016/j.tics.2019.04.005 31104816 PMC6876751

[desc13055-bib-0050] Rodebaugh, T. L. , Scullin, R. B. , Langer, J. K. , Dixon, D. J. , Huppert, J. D. , Bernstein, A. , …, Lenze, E. J. (2016). Unreliability as a threat to understanding psychopathology: The cautionary tale of attentional bias. Journal of Abnormal Psychology. 125(6):840–851. 27322741 10.1037/abn0000184 PMC4980228

[desc13055-bib-0051] Rodger, H. , Lao, J. , & Caldara, R. (2018). Quantifying facial expression signal and intensity use during development. Journal of Experimental Child Psychology. Elsevier Inc., 174, 41–59. 10.1016/j.jecp.2018.05.005 29906651

[desc13055-bib-0052] Sadeh, N. , & Bredemeier, K. (2011). Individual differences at high perceptual load: The relation between trait anxiety and selective attention. Cognition and Emotion, 25(4), 747–755. 10.1080/02699931.2010.500566 21547776 PMC3089738

[desc13055-bib-0053] Sato, W. , & Yoshikawa, S. (2007). Enhanced experience of emotional arousal in response to dynamic facial expressions. Journal of Nonverbal Behavior, 31(2), 119–135. 10.1007/s10919-007-0025-7

[desc13055-bib-0054] Schmukle, S. C. (2005). Unreliability of the dot probe task. European Journal of Personality, 10.1002/per.554

[desc13055-bib-0055] Serences, J. T. , Shomstein, S. , Leber, A. B. , Golay, X. , Egeth, H. E. , Yantis, S. (2005). Coordination of voluntary and stimulus‐driven attentional control in human cortex. Psychological Science, 16(2), 114–122. 10.1111/j.0956-7976.2005.00791.x 15686577

[desc13055-bib-0056] Soares, S. C. , Rocha, M. , Neiva, T. , Rodrigues, P. , Silva, C. F. (2015). Social anxiety under load: The effects of perceptual load in processing emotional faces. Frontiers in Psychology, 6, 1–6. 10.3389/fpsyg.2015.00479 25954232 PMC4404732

[desc13055-bib-0057] Spielberger, C. D. (1973). Manual for the State‐Trait Anxiety Inventory for Children. .

[desc13055-bib-0058] Spielberger, C. D. , & Gorsuch, R. L. (1983). State‐trait anxiety inventory for adults: sampler set: manual, test, scoring key. Mind Garden.

[desc13055-bib-0059] Springer, U. S. , Rosas, A. , McGetrick, J. , Bowers, D. (2007). Differences in startle reactivity during the perception of angry and fearful faces. Emotion, 7(3), 516–525. 10.1037/1528-3542.7.3.516 17683208

[desc13055-bib-0060] Sussman, T. J. , Heller, W. , Miller, G. A. , & Mohanty, A. (2013). Emotional distractors can enhance attention. Psychological Science, 24(11), 2322–2328. 10.1177/0956797613492774 24058065 PMC4408991

[desc13055-bib-0072] Theeuwes J. (2010). Top–down and bottom–up control of visual selection. Acta Psychologica, 135 (2), 77–99 20507828 10.1016/j.actpsy.2010.02.006

[desc13055-bib-0061] Theeuwes, J. , Kramer, A. F. , & Belopolsky, A. V. (2004). Attentional set interacts with perceptual load in visual search. Psychonomic Bulletin & Review, 11(4), 697–702. 10.3758/BF03196622 15581120

[desc13055-bib-0062] Van Bockstaele, B. , Verschuere, B. , Tibboel, H. , De Houwer, J. , Crombez, G. , & Koster, E. H. W. (2014). A review of current evidence for the causal impact of attentional bias on fear and anxiety. Psychological Bulletin, 10.1037/a0034834 24188418

[desc13055-bib-0063] Voss, A. , Nagler, M. , & Lerche, V. (2013). Diffusion models in experimental psychology: A practical introduction. Experimental Psychology, 10.1027/1618-3169/a000218 23895923

[desc13055-bib-0064] Voss, A. , & Voss, J. (2007). Fast‐dm: A free program for efficient diffusion model analysis. Behavior Research Methods, 10.3758/BF03192967 18183889

[desc13055-bib-0065] Voss, A. , Voss, J. , & Lerche, V. (2015). Assessing cognitive processes with diffusion model analyses: A tutorial based on fast‐dm‐30. Frontiers in Psychology, 6, 1–14. 10.3389/fpsyg.2015.00336 25870575 PMC4376117

[desc13055-bib-0066] White, C. N. , Ratcliff, R. , Vasey, M. W. , & McKoon, G. (2010). Anxiety enhances threat processing without competition among multiple inputs: A diffusion model analysis. Emotion, 10(5), 662–677. 10.1037/a0019474 21038949

[desc13055-bib-0067] Yantis, S. (2000). Goal‐directed and stimulus‐driven determinants of attentional control. Attention and Performance.

