# Supplementary material for: Computational modelling of attentional bias towards threat in paediatric anxiety
Source: Dev Sci. 2020 Nov 23;24(3):e13055. doi: 10.1111/desc.13055 (PMC8244064; doi:10.1111/desc.13055)
Supplement: Supplementary file 1 — Supplementary Material [file DESC-24-e13055-s001.docx]

**Supplementary material**

Table 1 Estimates for mixed effects models of accuracy and reaction time. Load and number of trials are within-participants factors and age is a between-participants factor. N = 60.

|  | Model summary | | | Confidence interval |  | Model comparison | |
| --- | --- | --- | --- | --- | --- | --- | --- |
|  | β | Std. Error | t/z value | LL | UL | X^2^ | *p* |
| ***Accuracy*** |  |  |  |  |  |  |  |
| *Fixed effects* |  |  |  |  |  |  |  |
| Intercept | 0.68 | 0.05 | 13.85 | 0.59 | 0.78 |  |  |
| Load | 0.60 | 0.05 | 12.20 | 0.50 | 0.69 | 75.53 | < .001*** |
| Age | 0.11 | 0.05 | 2.24 | 0.01 | 0.20 | 2.14 | .14 |
| Trial | -0.03 | 0.05 | -0.65 | -0.13 | 0.06 | .53 | .47 |
| Load*Age | -0.09 | 0.05 | -2.02 | -0.19 | -0.00 | 3.96 | < .05* |
|  |  |  |  |  |  |  |  |
| ***Reaction times*** |  |  |  |  |  |  |  |
| *Fixed effects* |  |  |  |  |  |  |  |
| Intercept | 889.65 | 40.62 | 21.90 | 810.04 | 969.27 |  |  |
| Load | -243.03 | 22.80 | -10.66 | -287.72 | -198.34 | 65.03 | < .001*** |
| Age | -145.02 | 35.36 | -4.10 | -214.33 | -75.72 | 23.70 | < .001*** |
| Trial | 16.59 | 31.31 | 0.53 | -44.78 | 77.97 | 0.28 | .59 |
| Load*Age | 9.73 | 21.24 | 0.46 | -31.89 | 51.35 | 0.18 | .67 |
|  |  |  |  |  |  |  |  |
| ***V*** |  |  |  |  |  |  |  |
| *Fixed effects* |  |  |  |  |  |  |  |
| Intercept | 0.97 | 0.09 | 11.37 | 0.80 | 1.14 |  |  |
| Load | 1.06 | 0.11 | 9.9 | 0.85 | 1.27 | 62.09 | < .001*** |
| Age | 0.25 | 0.09 | 2.88 | 0.08 | 0.42 | 10.00 | < .01** |
| Trial | -0.13 | 0.07 | -1.93 | -0.27 | 0.00 | 3.50 | .06 |
| Load*Age | -0.05 | 0.11 | -0.43 | -0.25 | 0.16 | 0.18 | 0.67 |
|  |  |  |  |  |  |  |  |
| ***T0*** |  |  |  |  |  |  |  |
| *Fixed effects* |  |  |  |  |  |  |  |
| Intercept | 0.43 | 0.02 | 17.88 | 0.38 | 0.48 |  |  |
| Load | -0.13 | 0.02 | -5.36 | -0.17 | -0.08 | 22.38 | < .001*** |
| Age | 0.02 | 0.02 | 0.77 | -0.03 | 0.07 | 0.04 | 0.84 |
| Trial | -0.02 | 0.02 | -0.84 | -0.06 | 0.02 | 0.70 | 0.40 |
| Load*Age | -0.05 | 0.02 | -1.99 | -0.09 | -0.00 | 3.82 | 0.05 |

* p < 0.05, ** p < 0.01, *** p < 0.001. The degrees of freedom for all model comparisons is 1. For accuracy and reaction time random effects are participant (intercept) and load (slope). For V and T0, the maximal model did not converge and therefore we included the random effect of subject (intercept) but removed load (slope).

Table 2 Means and standard deviations for reaction time (ms) and accuracy (%) by perceptual load, expression and load*expression

|  | Reaction time | Accuracy |
| --- | --- | --- |
| *Load* |  |  |
| High | 904.02 (514.54) | 74.58 (43.55) |
| Low | 655.48 (404.69) | 88.66 (31.72) |
|  |  |  |
| *Expression* |  |  |
| Fearful | 761.17 (468.89) | 80.91 (39.31) |
| Happy | 791.07 (488.49) | 82.19 (38.27) |
| Neutral | 788.40 (481.26) | 81.56 (38.79) |
|  |  |  |
| *High load only* |  |  |
| Fearful | 874.29 (508.62) | 74.49 (43.60) |
| Happy | 923.06 (520.84) | 74.87 (43.39) |
| Neutral | 911.24 (513.59) | 74.43 (43.63) |
|  |  |  |
| *Low load only* |  |  |
| Fearful | 651.77 (398.43) | 87.05 (33.58) |
| Happy | 650.47 (406.99) | 89.98 (30.04) |
| Neutral | 660.76 (407.82) | 88.96 (31.35) |

**Graphs showing the relationship between performance measures across the different facial expressions and the low load condition**

**
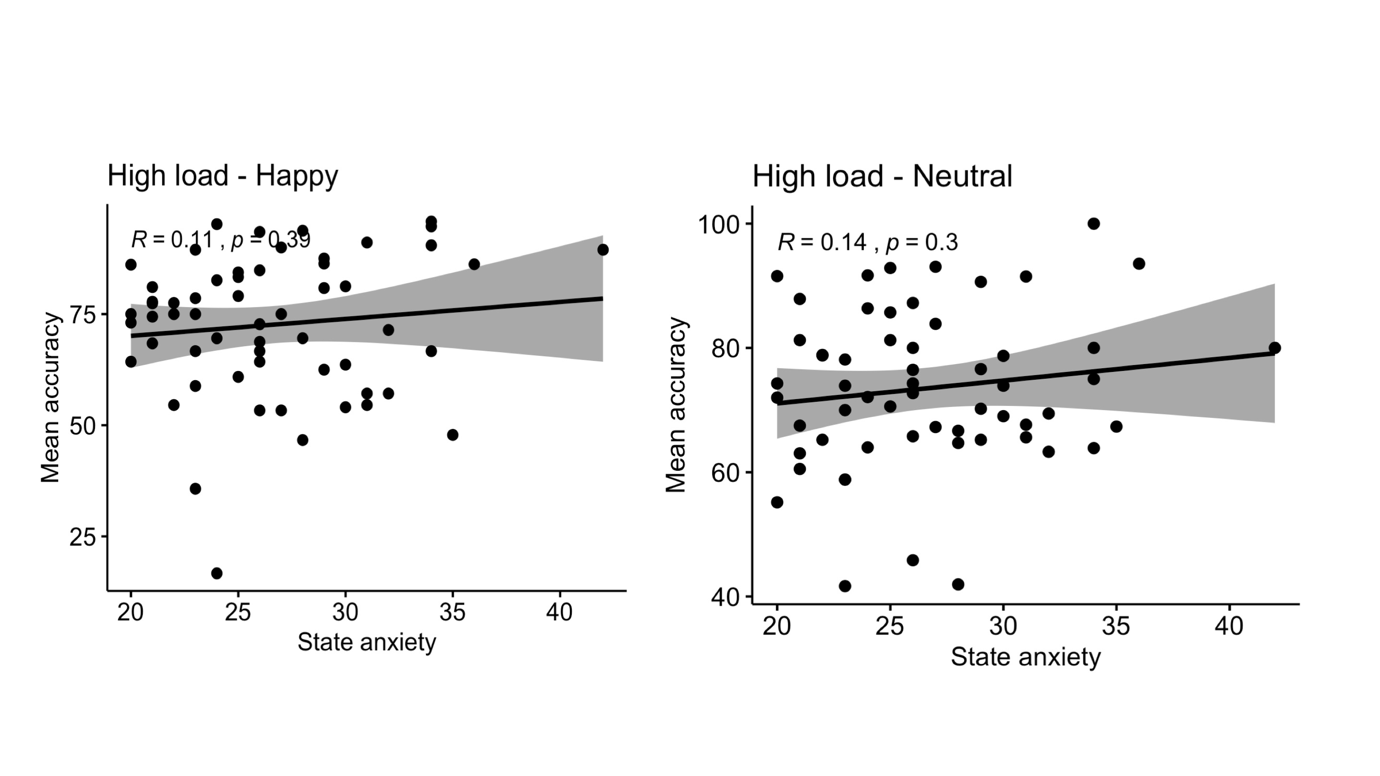
**

Supplementary Figure 1 Correlations between mean accuracy and state anxiety (high load condition)


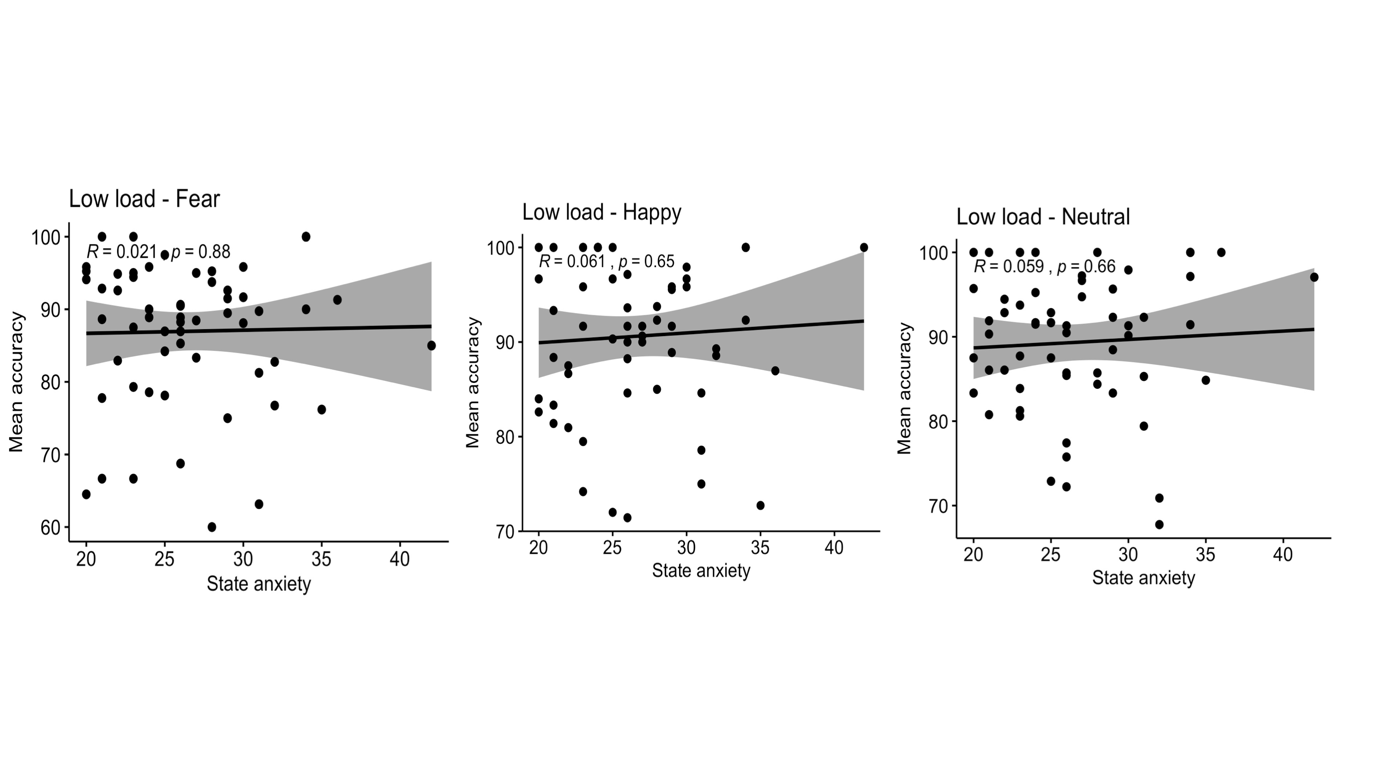


Supplementary Figure 2 Correlations between mean accuracy and state anxiety (low load condition)


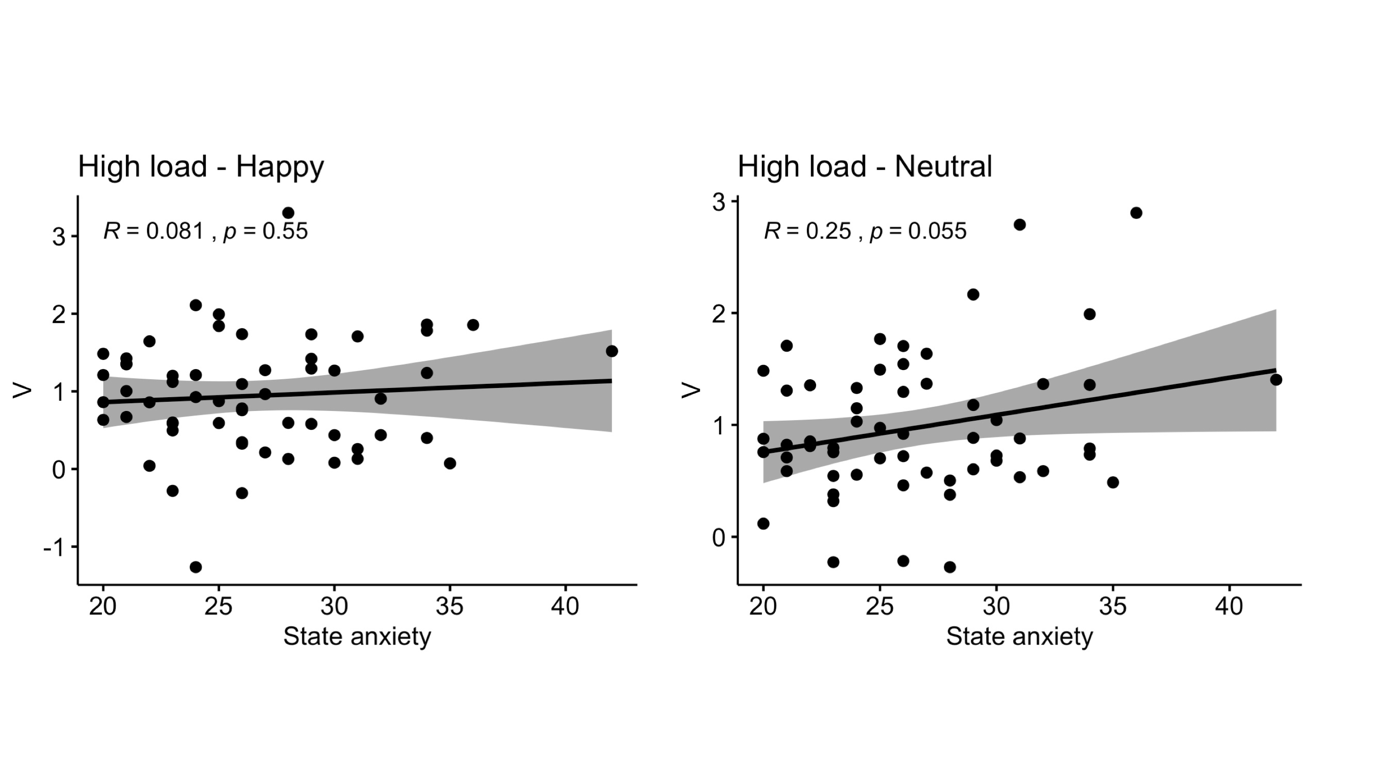


Supplementary Figure 3 Correlations between drift rate (V) and state anxiety (high load condition)


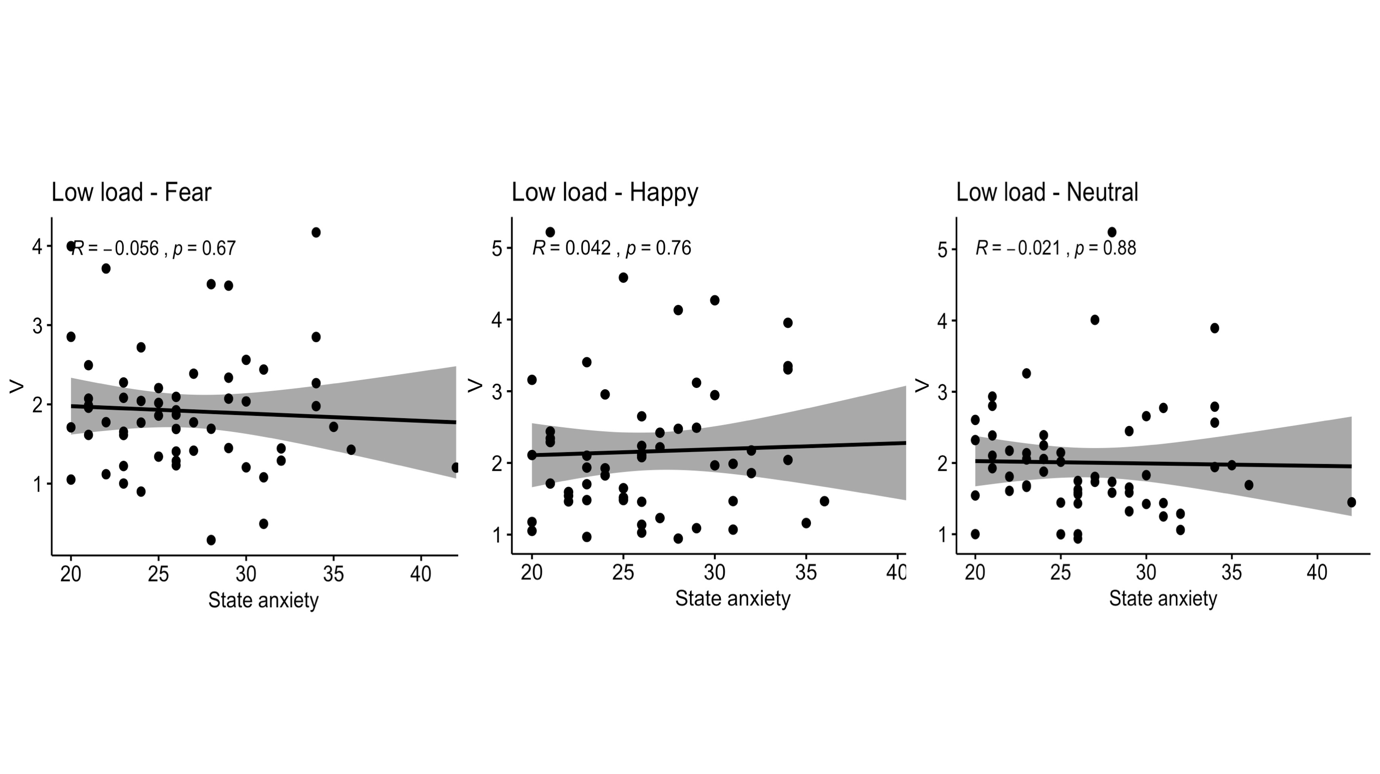


Supplementary Figure 4 Correlations between drift rate (V) and state anxiety (low load condition)

**Sensitivity Analyses**

***Results when minimum trial numbers included (trial numbers = 101, N = 60)***

Results presented in Supplementary Materials Table 3.

**Load**

*Accuracy*

There was a significant interaction between age and load (X2(1)=4.16, p = < .05) (Supplementary Material Table 3). Follow-up comparisons revealed a positive relationship between age and accuracy in the high load condition (r = .39, p = .002 CI [0.15 0.59]) but not in the low load condition (p > .05). The main effect of load was significant (X2(1)=70.06, p = <.001), with significantly higher accuracy in the low load (M = 0.87; SD = .33) compared to the high load condition (M = 0.73; SD = .44). The main effect of age was significant (X2(1)=4.73, p = .03), reflecting that children performed with higher accuracy with increasing age.

*Reaction times*

The interaction between age and load was not significant. There was a significant main effect of load (X^2^(1)=66.32, p = <.001), with significantly higher reaction times in the high load (*M* = 899.81; SD = 520.32) condition compared with the low load (*M* = 626.94; SD = 386.57) condition. There was also a significant main effect of age (X^2^(1)=13.32, p = <.001), reflecting that children responded faster with increasing age.

*Drift diffusion model measures*

The main effect of load on *V* was significant (X^2^(1)=45.75, p = <.001), with significantly higher *V* in the low load condition (*M* = 1.97; SD = 0.74) than in the high load condition (*M* = 0.98; SD = 0.75). There was a significant main effect of age on *V* (X^2^(1)=5.47, p = .02), reflecting that *V* increases with age in both load conditions. There was a significant interaction between age and load on t0 (X^2^(1)=4.06, p < .05). Follow-up comparisons revealed a negative relationship between age and t0 in the low load condition (r = -.26, p < .05 CI [-0.48, -0.00]) but not in the high load condition (p > .05). There was also a significant main effect of load on t0 (X^2^(1)=23.79, p < .001), with significantly higher t0 in the high load (*M* = 0.43; SD = 0.22) compared with the low load condition (*M* = 0.30; SD = 0.15). No other main effects or interactions were significant.

**Relationship between load, emotional distractors and anxiety**

*Accuracy*

There was a positive correlation between mean accuracy and state anxiety scores on trials where fearful faces were presented in the high load condition, (r = .31, p = .02 CI [0.06 0.52]), but not in the low load condition (r = .08, p > .05 CI [-0.18 0.33]). This remained significant when age (r = .34, p = .01 CI [0.09 0.55]) and performance on the fearful face trials of the low load condition were included as covariates (r = .33, p = .01 CI [0.08 0.54]). This finding was specific to fearful faces: there were no significant correlations between state anxiety and accuracy performance on either the happy or neutral trials. To further investigate the specificity of this relationship we also conducted a partial correlation controlling for accuracy in the low load fearful face trials and accuracy on the other facial expression conditions (high load), as well as number of trials, and the correlation remained significant (r = .27, p = .048 CI [0.02 0.49]).

*Reaction times*

There were no significant correlations between state anxiety and RT in any condition.

*Drift diffusion modelling*

It was not possible to conduct the drift diffusion analysis because of the low numbers of trials.

Table 3 Estimates for mixed effects models of accuracy and reaction time when minimum trial number is included across all participants. Load and number of trials are within-participants factors and age is a between-participants factor. N = 60.

|  | Model summary | | | Confidence interval |  | Model comparison | |
| --- | --- | --- | --- | --- | --- | --- | --- |
|  | β | Std. Error | t/z value | LL | UL | X^2^ | *p* |
| ***Accuracy*** |  |  |  |  |  |  |  |
| *Fixed effects* |  |  |  |  |  |  |  |
| Intercept | 0.63 | 0.04 | 14.29 | 0.55 | 0.72 |  |  |
| Load | 0.61 | 0.05 | 11.24 | 0.50 | 0.72 | 70.06 | < .001*** |
| Age | 0.12 | 0.04 | 2.63 | 0.03 | 0.20 | 4.73 | .03* |
| Trial | -0.02 | 0.05 | -0.52 | -0.12 | 0.07 | 0.32 | .57 |
| Load*Age | -0.11 | 0.05 | -2.04 | -0.21 | -0.00 | 4.16 | .04* |
|  |  |  |  |  |  |  |  |
| ***Reaction times*** |  |  |  |  |  |  |  |
| *Fixed effects* |  |  |  |  |  |  |  |
| Intercept | 878.92 | 41.20 | 21.33 | 798.17 | 959.67 |  |  |
| Load | -257.96 | 23.59 | -10.94 | -304.20 | -211.72 | 66.32 | < .001*** |
| Age | -107.85 | 36.91 | -2.79 | -175.19 | -30.50 | 13.32 | < .001*** |
| Trial | 2.32 | 28.01 | 0.08 | -52.57 | 57.21 | 0.01 | .93 |
| Load*Age | 5.38 | 21.98 | 0.25 | -37.70 | 48.47 | 0.06 | .81 |
|  |  |  |  |  |  |  |  |
| ***V*** |  |  |  |  |  |  |  |
| *Fixed effects* |  |  |  |  |  |  |  |
| Intercept | 0.98 | 0.09 | 10.46 | 0.79 | 1.16 |  |  |
| Load | 1.00 | 0.11 | 7.62 | 0.74 | 1.25 | 45.75 | < .001*** |
| Age | 0.26 | 0.10 | 2.71 | 0.07 | 0.45 | 5.47 | .02** |
| Trial | -0.02 | 0.07 | -0.31 | -0.16 | 0.12 | 0.09 | .76 |
| Load*Age | -0.18 | 0.13 | -1.41 | -0.44 | 0.07 | 0.16 | 0.16 |
|  |  |  |  |  |  |  |  |
| ***T0*** |  |  |  |  |  |  |  |
| *Fixed effects* |  |  |  |  |  |  |  |
| Intercept | 0.43 | 0.02 | 18.24 | 0.38 | 0.48 |  |  |
| Load | -0.13 | 0.02 | -5.60 | -0.18 | -0.09 | 23.79 | < .001*** |
| Age | 0.02 | 0.02 | 0.79 | -0.03 | 0.07 | 0.04 | 0.83 |
| Trial | -0.02 | 0.02 | -0.81 | -0.06 | 0.02 | 0.65 | 0.42 |
| Load*Age | -0.05 | 0.02 | -2.05 | -0.09 | -0.00 | 4.06 | 0.04* |

* p < 0.05, ** p < 0.01, *** p < 0.001. The degrees of freedom for all model comparisons is 1. For accuracy and reaction time random effects are participant (intercept) and load (slope). For V and T0, the maximal model did not converge and therefore we included the random effect of subject (intercept) but removed load (slope).

***Results for children who were excluded (N = 48)***

Results presented in Supplementary Materials Table 4. Total number of trials experienced by participants varied (range = 100-516 trials, median = 191, mean = 227.17, standard deviation = 107.78).

**Load**

*Accuracy*

The main effect of load was significant (X2(1)=15.11, p = <.001), with significant higher accuracy in the low load (M = 0.62; SD = .48) compared to the high load condition (M = 0.53; SD = .50). The main effect of age approached significance (X2(1)=3.53, p = .06). No other main effects or interactions were significant.

*Reaction times*

There was a significant main effect of age (X^2^(1)=21.90, p = <.001), reflecting that children responded faster with increasing age. No other main effects or interactions were significant.

*Drift diffusion model measures*

The main effect of load on *V* was significant (X2(1)=5.67, p = .02), with significantly higher *V* in the low load (M = 0.37; SD = .61) compared to the high load condition (M = 0.10; SD = .59). There was a significant main effect of age on t0 (X^2^(1)=10.01, p = .002), reflecting that t0 decreased with increasing age. No other main effects or interactions were significant.

**Relationship between load, emotional distractors and anxiety**

Of the 48 children included here, 4 did not complete the STAI-C due to time constraints during testing, therefore the number of participants included in this analysis was 44.

*Accuracy*

There were no significant correlations between state anxiety and accuracy in any condition.

*Reaction times*

There were no significant correlations between state anxiety and RT in any condition.

*Drift diffusion modelling*

It was not possible to conduct the drift diffusion analysis because of the low numbers of trials.

Table 4 Estimates for mixed effects models of accuracy and reaction time for participants that did not reach 67% accuracy. Load and number of trials are within-participants factors and age is a between-participants factor. N = 48.

|  | Model summary | | | Confidence interval |  | Model comparison | |
| --- | --- | --- | --- | --- | --- | --- | --- |
|  | β | Std. Error | t/z value | LL | UL | X^2^ | *p* |
| ***Accuracy*** |  |  |  |  |  |  |  |
| *Fixed effects* |  |  |  |  |  |  |  |
| Intercept | 0.07 | 0.02 | 2.66 | 0.02 | 0.11 |  |  |
| Load | 0.22 | 0.05 | 4.35 | 0.12 | 0.31 | 15.11 | < .001*** |
| Age | 0.04 | 0.02 | 1.71 | -0.01 | 0.09 | 3.53 | .06 |
| Trial | 0.05 | 0.02 | 2.43 | 0.01 | 0.10 | 5.14 | .02* |
| Load*Age | -0.01 | 0.05 | -0.23 | -0.10 | 0.08 | 0.05 | .82 |
|  |  |  |  |  |  |  |  |
| ***Reaction times*** |  |  |  |  |  |  |  |
| *Fixed effects* |  |  |  |  |  |  |  |
| Intercept | 477.22 | 34.92 | 13.67 | 408.78 | 545.65 |  |  |
| Load | -31.35 | 23.62 | -1.33 | -77.65 | 14.95 | 1.72 | .19 |
| Age | -87.30 | 20.26 | -4.31 | -127.02 | -47.59 | 21.90 | < .001*** |
| Trial | -31.15 | 37.49 | -0.83 | -104.62 | 42.32 | 0.67 | .41 |
| Load*Age | 11.46 | 20.11 | 0.57 | -27.95 | 50.87 | 0.32 | .57 |
|  |  |  |  |  |  |  |  |
| ***V*** |  |  |  |  |  |  |  |
| *Fixed effects* |  |  |  |  |  |  |  |
| Intercept | 0.10 | 0.09 | 1.13 | -0.07 | 0.27 |  |  |
| Load | 0.28 | 0.11 | 2.50 | 0.06 | 0.51 | 5.67 | .02* |
| Age | -0.02 | 0.09 | -0.18 | -0.19 | 0.16 | 0.26 | .61 |
| Trial | 0.06 | 0.07 | 0.78 | -0.09 | 0.20 | 0.62 | .43 |
| Load*Age | 0.10 | 0.11 | 0.89 | -0.13 | 0.32 | 0.79 | 0.37 |
|  |  |  |  |  |  |  |  |
| ***T0*** |  |  |  |  |  |  |  |
| *Fixed effects* |  |  |  |  |  |  |  |
| Intercept | 0.13 | 0.00 | 30.31 | 0.12 | 0.14 |  |  |
| Load | 0.00 | 0.01 | 1.22 | -0.00 | 0.02 | 1.34 | 0.25 |
| Age | -0.01 | 0.00 | -1.96 | -0.02 | -0.00 | 10.01 | 0.002** |
| Trial | -0.00 | 0.00 | -0.76 | -0.01 | 0.00 | 0.65 | 0.42 |
| Load*Age | -0.01 | 0.01 | -1.17 | -0.02 | 0.00 | 1.36 | 0.24 |

* p < 0.05, ** p < 0.01, *** p < 0.001. The degrees of freedom for all model comparisons is 1. For accuracy and reaction time random effects are participant (intercept) and load (slope). For V and T0, the maximal model did not converge and therefore we included the random effect of subject (intercept) but removed load (slope).

**Correlations between drift diffusion model parameters and canonical measures**


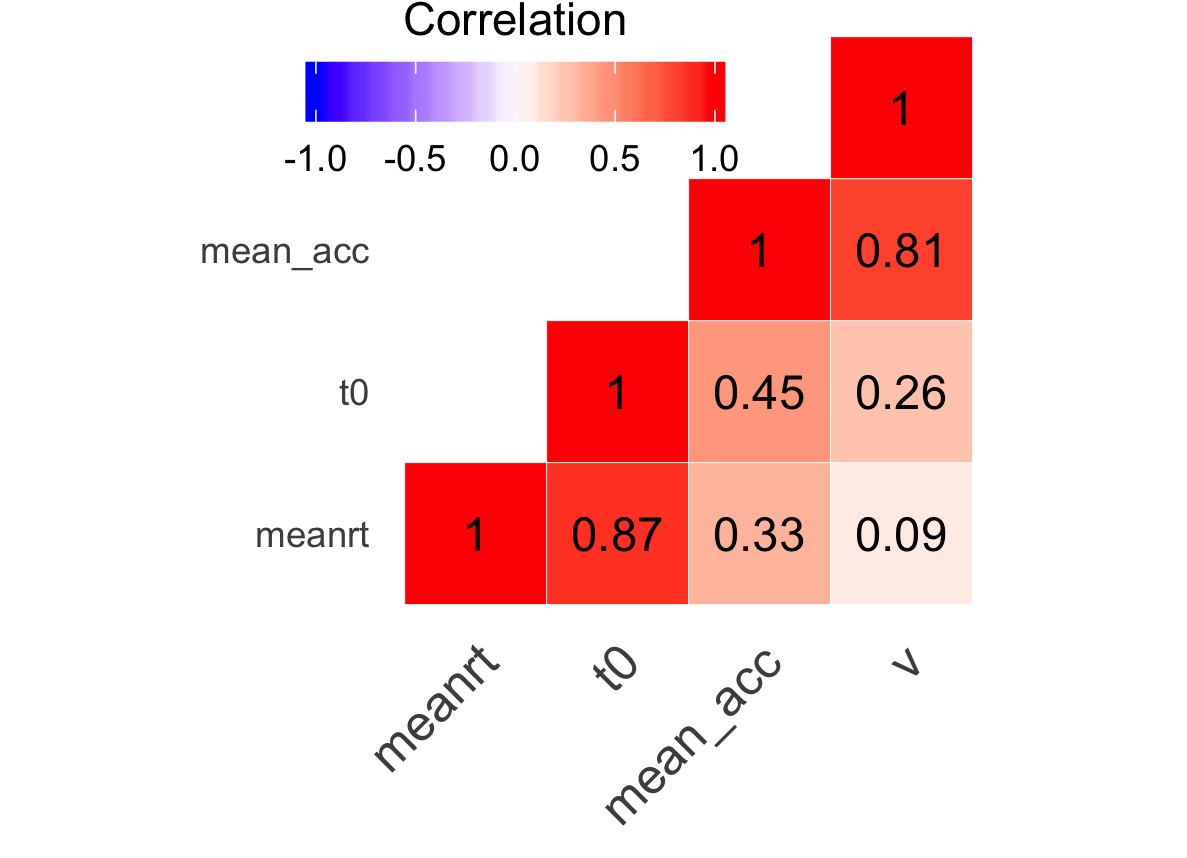


Supplementary Figure 5 Correlation heat-map between drift diffusion model parameters (drift rate, V and non-decisional processes, t0) and canonical measures (mean response time and accuracy), for the high load condition. Pearson’s correlation coefficient included.


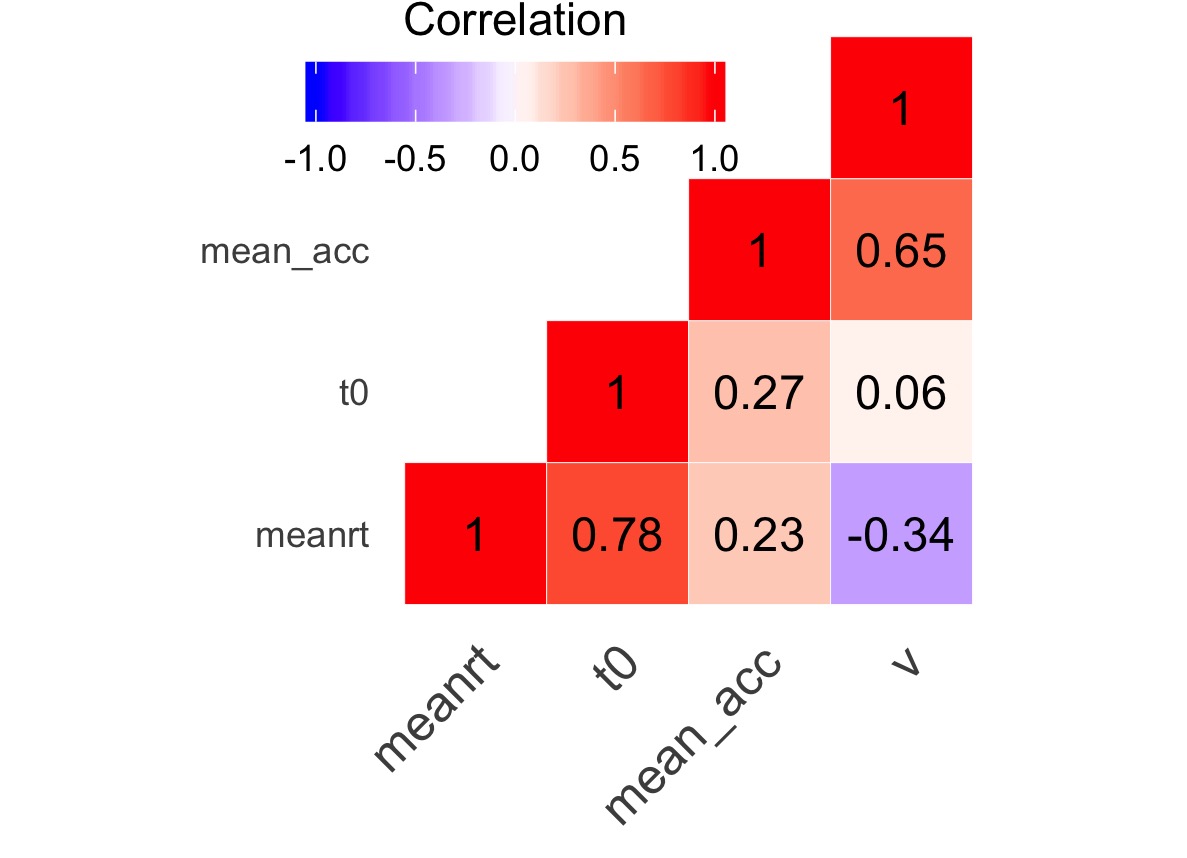


Supplementary Figure 6 Correlation heat-map between drift diffusion model parameters (drift rate, V and non-decisional processes, t0) and canonical measures (mean response time and accuracy), for the low load condition. Pearson’s correlation coefficient included.
